# Transplant or dialysis: What’s the better choice for RCC-induced ESRD patients? A 20-year analysis of OPTN/UNOS data

**DOI:** 10.3389/fonc.2022.955771

**Published:** 2022-09-29

**Authors:** Xiaowei Hao, Wenhui Lai, Xinze Xia, Junnan Xu, Yangyang Wu, Chao Lv, Kaikai Lv, Shuai Huang, Zhenjun Luo, Qingyang Meng, Qing Yuan, Jun Dong

**Affiliations:** ^1^ Department of Urology, The Third Medical Center, Chinese PLA General Hospital, Beijing, China; ^2^ Department of Urology, No.971 Hospital of PLA Navy, Tsingtao, Shandong, China; ^3^ Department of Postgraduate, Hebei North University, Zhangjiakou, China; ^4^ Department of Urology, Shanxi Medical University, Taiyuan, China; ^5^ Affiliated Hospital of Weifang Medical University, School of Clinical Medicine, Weifang Medical University, Weifang, China

**Keywords:** renal cell carcinoma, kidney transplantation, end-stage renal disease, UNOS/OPTN, propensity score match

## Abstract

**Purpose:**

The incidence of end-stage renal disease (ESRD) caused by renal cell carcinoma (RCC) is increasing with the high prevalence of RCC as well as those with treatment-related renal function impairment. Worries about tumor recurrence after transplant-related immunosuppression hinder the recommendation of kidney transplantation for RCC-induced ESRD patients. However, no direct analysis has been performed to identify whether kidney transplantation can offer better survival than maintaining dialysis.

**Materials and methods:**

This retrospective population-based cohort study was based on Organ Procurement and Transplantation Network data released in March 2021. Characteristics and outcomes were compared, including the patient and graft survival of candidates and recipients with RCC-induced ESRD etiology as well as other primary diseases.

**Results:**

Patients with RCC-induced ESRD were older; more likely to be male, White, and obese; and more likely to have a history of diabetes and dialysis. They also had higher creatinine levels, more delayed graft function, more primary non-function, and higher Kidney Donor Profile Index score donors, compared with the glomerulonephritis (GN) group. While waiting, RCC candidates suffered the worst outcomes of all groups, a 44% (adjusted hazard ratio [aHR], 1.44 [1.27–1.62]) higher risk of removal than GN patients. After transplantation, RCC recipients demonstrated comparable patient survival and better graft survival (p=0.21 and p=0.13, respectively). Compared with still-waiting RCC patients, the RCC recipients who received kidney transplants had significantly better outcomes (13.6 [9.3–17.8] vs. 61 [52–68.4] %), decreasing the death or deteriorating risk by 84% (aHR, 0.16 [0.13–0.20]).

**Conclusions:**

Patients with RCC-induced ESRD can dramatically benefit from kidney transplantation. Hence, these patients should not be limited to transplantation by strict strategies or a delayed waiting time out of their malignancy history.

## Introduction

Renal cancers, accounting for approximately 3%–5% of all adult malignancies, are the sixth most common cancer in men and the ninth in women, with a total of 75,000 new cases reported in 2021 in the United States (1). Among them, renal cell carcinoma (RCC) leads to approximately 85%–95% of renal cancers (2, 3). Despite efforts to improve the early detectable rate and effective treatment help patients’ overall survival and renal function maintenance, several multicenter analyses in the United States and Europe demonstrated that roughly 2% of T1a renal tumor cases with normal preoperative renal function will develop end-stage renal disease (ESRD) in the first 10 years after nephron-sparing surgery (4–7). In terms of renal functional outcomes, thermal ablation is similar with partial nephrectomy (8–10). Moreover, tumors larger than T1a requiring radical nephrectomy are generally subjected to worse renal function decreases. Regarding advanced tumor, huge tumor or tumor thrombus usually devasts tumor side kidney; meanwhile, systemic antitumor therapy for metastatic RCC including targeted therapies and immunotherapy is also associated with renal toxicity. The prevalence of RCC-induced ESRD continues to increase because more RCC patients are successfully treated and achieve a 10-year survival rate exceeding 80%. Even if they are forced to start dialysis, patients might consider kidney transplantation (KTX) provided that the cancer has been cured (11, 12). A high rate (7%) of pretransplant malignancy (pre-TM) in all solid organ transplant recipients is expected to increase with the expansion of eligibility criteria to patients (13).

However, pre-TM is considered a relative contraindication because pre-TM itself is a risk factor for cancer recurrence and renal cancer recurs in up to 21% of cases (14) as well because immunosuppressive therapies throughout a recipient’s life contribute to the risk of cancer onset and recurrence (15). Although some guidelines on KTX for RCC-induced ESRD have been reported, few studies have reported the exact number of RCC-induced ESRD patients who successfully received transplantation (16).

Hence, whether KTX for RCC-induced ESRD patients can provide better survival than maintaining dialysis remains to be elucidated. To improve our understanding, this long-term population-based cohort study examined patients with RCC-induced ESRD registered in the Organ Procurement and Transplantation Network (OPTN). This is the first study to systematically analyze the characteristics and outcomes of RCC-induced ESRD patients on the national scale to evaluate the overall transplant management strategy in terms of life expectancy and potential risk for RCC-induced ESRD patients.

## Methods

### Data source, study design, and participants

**Figure 1:**
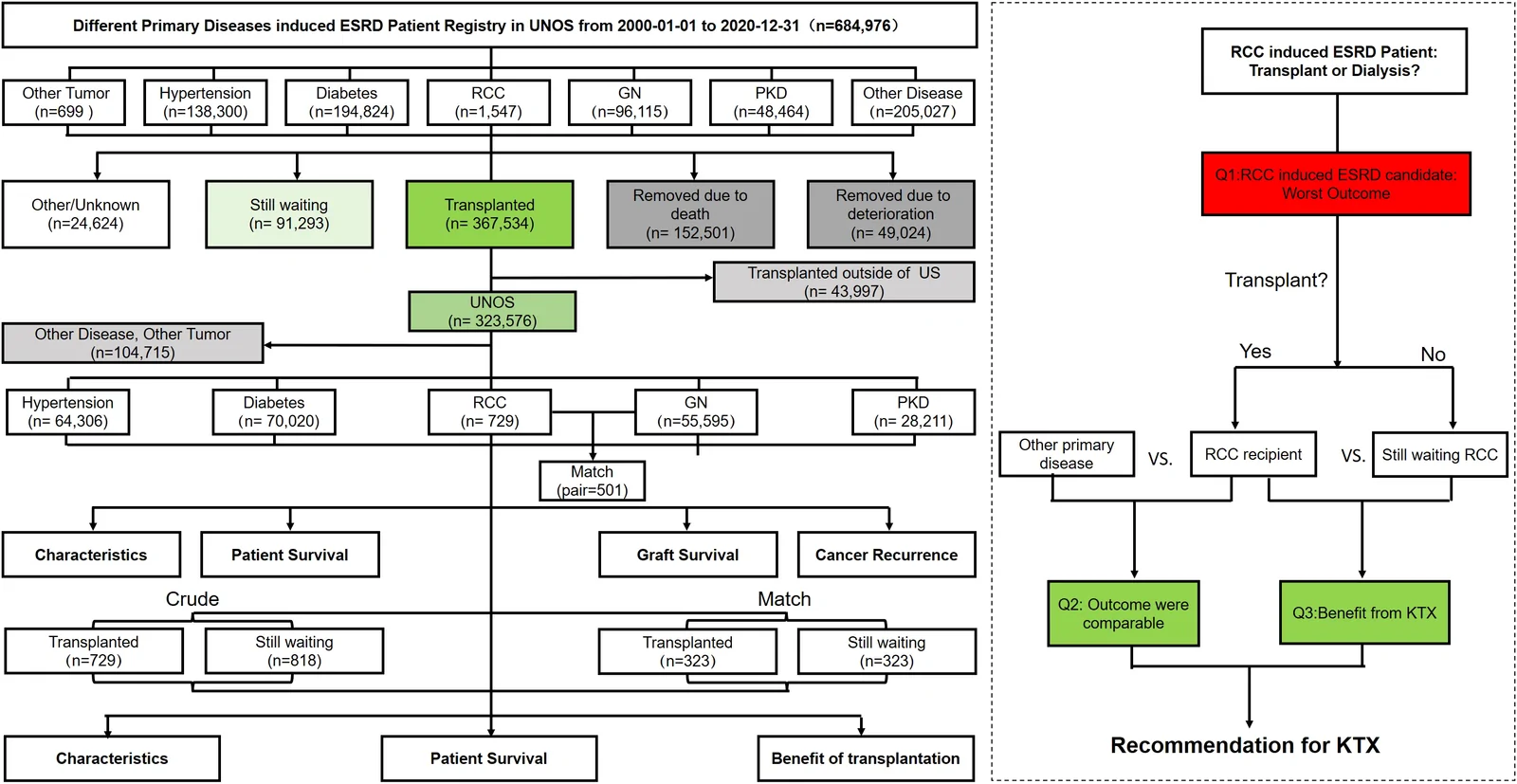
Flowchart of the study cohort enrollment process.

This retrospective population-based cohort study analyzed the KTX candidates whose transplantation data were registered in the OPTN Standard Transplant Analysis and Research file released in March 2021. The patients were grouped by “primary disease diagnosis at the time of listing” into RCC, other tumor, glomerulonephritis (GN), polycystic kidney disease (PKD), diabetes, hypertension, and other disease-induced ESRD groups. Other tumors included myeloma (n=344), Wilms tumor (n=272), lymphoma (n=42), and incidental carcinoma (n=41). Other ESRD primary diseases included familial disease and autoimmune disease. To note, considering the sample size and space limitations, the other tumors and other primary disease groups were analyzed further but not listed in the results (**Figure 1**).

### Exposure and outcome classification and assessment

The time to outcomes of the candidate was defined as the date when a candidate was added to the transplant waiting list until the date of the outcome (transplant, removed due to death or deterioration, or the end of the study period). The time to outcomes of the recipient was defined as the date when the recipient received transplantation until the date of the outcome (patient death or graft failure), censored for loss to follow-up, or the end of the study period. Outcomes are indicated by two sides, i.e., the patient status at the follow-up end while the other side was candidate removal incidence, recipients’ patient survival (PS), graft survival (GS), and death-censored GS (DCGS). Five-year survival or incident rate was used as the standard in this study.

### Statistical analysis

The patients’ demographic and clinical characteristics were compared using the chi-square test for categorical variables and Student’s t-test for continuous variables. Survival analysis is presented as Kaplan–Meier curves and was compared using log-rank tests. GN and PKD were selected as the control group since they showed favorable outcomes. Propensity score matching (PSM) was used to eliminate the baseline confounders between groups. Factors such as age, ethnicity, and sex exhibited obvious disparities between the matched groups and were set as exact matching variables. A Cox model was adopted to show the candidate removal risk and extent of transplantation benefit.

The logit of the propensity score match was nearest neighbor matching with a 1:1 ratio, without replacement, and with a caliper width equal to 0.2 of the standard deviation. All analyses were performed using RStudio software version 1.1.456. P values <.05 were considered statistically significant, and all confidence intervals (CIs) used a 95% threshold. Descriptive statistics were used to summarize and present the data.

## Results

From January 2000 to December 2020, 684,976 patients registered in United Network for Organ Sharing (UNOS) applied for admission to KTX, among whom, a total of 367,534 patients received KTX, of which 1,547 candidates and 729 recipients were registered as RCC-induced ESRD.

### Candidates’ characteristics and outcomes

Among the candidate cohorts, the RCC group was the oldest (60.26 [10.60] years) and had a significantly higher proportion of patients who were men (75.5%), were White (67.0%), had a diabetes history (19.0%), had a body mass index (BMI) ≥30 kg/m^2^ (33.9%), had a college or postgraduate degree (53.3%), and had a dialysis history (73.1%), while a lower proportion of patients had primary health insurance (37%) (**Table 1**). Approximately 11.5% of the RCC candidates were still waiting for transplantation and 54.2% had received transplants; both ranked third lowest in all groups. The transplant rate of RCC candidates was similar to that of the non-cancer population (53.7%). Moreover, the transplant rate for other tumors were myeloma (62.5% [n=215]), Wilms tumor (68.8% [n=187]), lymphoma (64.3% [n=27]), and incidental carcinoma (58.5% [n=24]). Twelve percent (n=185) of RCC candidates were removed due to deterioration, a proportion that was the highest among all groups (**Table 2**).

**Table 1:** Candidates’ characteristics.

Characteristics		RCC(n = 1,547)	GN(n = 96,115)	PKD(n = 48,464)	Diabetes(n = 194,824)	Hypertension(n = 138,300)	P-value
**Age, yrs, mean (SD)**		60.26 (10.60)	44.93 (15.13)	52.31 (11.33)	56.00 (10.22)	52.47 (13.01)	<.001
**Sex, M, No.(%)**		1,168 (75.5)	58,502 (60.9)	25,854 (53.3)	127,344 (65.4)	90,919 (65.7)	<.001
**Ethnicity, No.(%)**	**White**	1,037 (67.0)	48,789 (50.8)	34,949 (72.1)	73,146 (37.5)	42,520 (30.7)	<.001
	**Black**	350 (22.6)	21,522 (22.4)	6,122 (12.6)	55,766 (28.6)	65,654 (47.5)	<.001
	**Hispanic**	116 (7.5)	14,334 (14.9)	5,008 (10.3)	45,305 (23.3)	19,862 (14.4)	<.001
**BMI ≥ 30 kg/m^2^, Y, No.(%)**		604 (39.0)	31,954 (33.2)	15,371 (31.7)	93,657 (48.1)	50,833 (36.8)	<.001
**Diabetes, Y, No.(%)**		294 (19.0)	8,308 (8.6)	3,228 (6.7)	194,522 (99.8)	22,774 (16.5)	<.001
**Education level, college degree, No.(%)**		825 (53.3)	49,371 (51.4)	27,535 (56.8)	87,870 (45.1)	57,633 (41.7)	<.001
**Private health insurance, No.(%)**		573 (37.0)	51,546 (53.6)	30,148 (62.2)	76,071 (39.0)	50,162 (36.3)	NaN
**Working for income, Y, No.(%)**		375 (24.2)	35,980 (37.4)	21,503 (44.4)	43,686 (22.4)	35,236 (25.5)	<.001
**Dialysis, Y, No.(%)**		1,131 (73.1)	60,669 (63.1)	22,111 (45.6)	149,054 (76.5)	107,183 (77.5)	<.001

BMI, body mass index; GN, glomerulonephritis; M, male; PKD, primary kidney disease; RCC, renal cell carcinoma; SD, standard deviation; Y, yes; yrs, years.

**Table 2:** Candidates’ status and cause of death by primary disease.

**Candidate status, No.(%)**	**RCC** **(n = 1,547)**	**GN** **(n = 96,115)**	**PKD** **(n = 48,464)**	**Diabetes** **(n = 194,824)**	**Hypertension** **(n = 138,300)**	**P-value**
**Still waiting**	178 (11.5)	11,438 (11.9)	5,822 (12.0)	33,194 (17.0)	17,829 (12.9)	<.001
**Transplanted**	838 (54.2)	64,036 (66.6)	33,103 (68.3)	78,230 (40.2)	73,544 (53.2)	<.001
**Removed due to deterioration**	185 (12.0)	3,537 (3.7)	1,789 (3.7)	21,236 (10.9)	9,908 (7.2)	<.001
**Died of cardiocerebrovascular infection**	34 (2.2)	1,186 (1.2)	513 (1.1)	8,356 (4.3)	3,555 (2.6)	<.001
**Died of infection**	9 (0.6)	400 (0.4)	175 (0.4)	2,177 (1.1)	884 (0.6)	<.001
**Died of cancer**	12 (0.8)	116 (0.1)	58 (0.1)	310 (0.2)	232 (0.2)	<.001
**Other/Unknown**	291 (18.8)	15,402 (16.0)	7,004 (14.5)	51,321 (26.3)	32,348 (23.4)	<.001

GN, glomerulonephritis; PKD, primary kidney disease; RCC, renal cell carcinoma.

**Figure 2:**
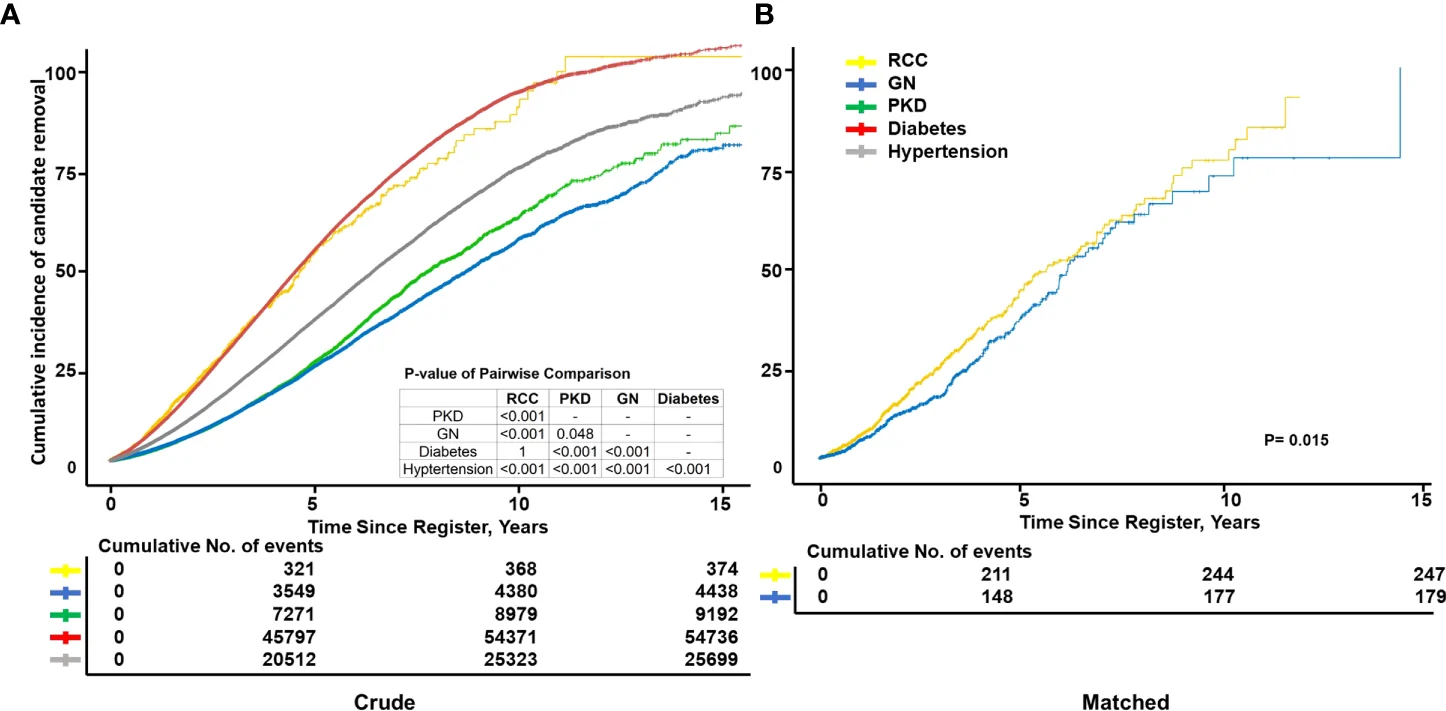
Cumulative incidence of candidate removal. Kaplan–Meier curves showing the cumulative incidence of candidate removal due to death and deterioration, including those caused by different primary diseases before transplantation **(A)** as well as end-stage renal disease (ESRD) caused by renal cell carcinoma (RCC), glomerulonephritis (GN), and primary kidney disease (PKD) after propensity score matching (n=1,312). **(B)** Exact matching variables: Age, sex, ethnicity, diabetes history, and education level; not exact: Body mass index, primary insurance, and income.

There was a significant difference in survival among the five candidate groups except for the RCC group parallel with the diabetes group (log-rank test; P=1). The 5-year removal incidence rate ranking from high to low was diabetes (47.4 [47.0–47.8]%), RCC (46.9 [42.2–51.2]%), hypertension (31.7 [31.3–32.7]%), PKD (22.0 [21.2–22.8]%), and GN (21.1 [20.6–21.6]%) (**Figure 2A**). After exact PSM with GN, the RCC group still had poorer outcomes, of which the 5-year cumulative removal incidence was 44% higher than that of the GN group (aHR, 1.44 [1.27–1.62], 44.5 [39.0–49.5] vs. 36.6 [30.7–42.1] %, p=0.015) (**Figure 2B**). Similarly, the outcome of RCC group was also poorer than PKD group after matching (aHR,1.71[1.51-1.94], 5-year cumulative removal incidence rate, 43.2 [36.8–49.0] vs.28.3 [22.1–34.0] %, p<0.001) (**Supplementary Figure 1A**).

### Recipients’ characteristics and outcomes

After transplantation, the distribution of recipient demographic characteristics was similar to those of the candidates. The RCC group underwent a relatively longer dialysis duration before transplantation (3.79 [3.03] years), had a significantly poorer recent kidney function (creatine, 2.12 [1.53] mg/dl), and were more vulnerable to delayed graft function (23.4%) and primary non-function (3.3%) (**Table 3**).

**Table 3:** Demographic and clinical characteristics of the recipients and donors.

Characteristics		RCC(n = 729)	GN(n=55,595)	PKD(n = 28,211)	Diabetes(n = 70,020)	Hypertension(n = 64,306)	P- value
**Age, yrs, mean (SD)**		60.74 (11.16)	44.92 (15.42)	52.99 (11.58)	57.58 (10.03)	52.95 (13.15)	<.001
**Sex, M, No.(%)**		549 (75.3)	20 (80.0)	93 (53.4)	13 (59.1)	15,037 (53.3)	<.001
**Ethnicity, No.(%)**	**White**	509 (69.8)	29,815 (53.6)	21,440 (76.0)	29,225 (41.7)	20,987 (32.6)	<.001
	**Black**	158 (21.7)	11,254 (20.2)	2,991 (10.6)	19,378 (27.7)	29,122 (45.3)	
	**Hispanic**	46 (6.3)	8,426 (15.2)	2,645 (9.4)	14,721 (21.0)	9,677 (15.0)	
**BMI ≥ 30 kg/m^2^, Y, No.(%)**		247 (33.9)	16,807 (30.2)	8,230 (29.2)	32,402 (46.3)	21,986 (34.2)	<.001
**Creatinine most recent, mg/dl, mean (SD)**		2.12 (1.53)	1.95 (1.39)	1.66 (1.08)	1.90 (1.19)	2.07 (1.52)	<.001
**Dialysis duration, yrs, mean (SD)**		3.79 (3.03)	2.87 (3.12)	2.28 (2.81)	3.46 (2.86)	4.07 (3.55)	<.001
**ABO compatible, Y, No.(%)**		723 (99.2)	55,029 (99.0)	27,935 (99.0)	69,230 (98.9)	63,687 (99.0)	.029
**Multiple organ transplant, Y, No.(%)**		24 (3.3)	791 (1.4)	571 (2.0)	2,054 (2.9)	791 (1.2)	<.001
**HLA mismatch ≥ 3, Y, No.(%)**		597 (81.9)	44,509 (80.1)	23,138 (82.0)	58,645 (83.8)	54,822 (85.3)	<.001
**PRA ≥ 30%, Y, No.(%)**		111 (15.2)	10,533 (18.9)	5,215 (18.5)	12,171 (17.4)	12,049 (18.7)	<.001
**Transplantation history, Y, No.(%)**		29 (4.0)	4,390 (7.9)	985 (3.5)	1,883 (2.7)	2,527 (3.9)	<.001
**Transfusion history, Y, No.(%)**		93 (12.8)	5,728 (10.3)	2,200 (7.8)	7,323 (10.5)	6,871 (10.7)	<.001
**Diabetes, Y, No.(%)**		97 (13.3)	3,762 (6.8)	1,523 (5.4)	69,894 (99.8)	8,541 (13.3)	<.001
**Primary non-function, Y, No.(%)**		25 (3.4)	1,411 (2.5)	637 (2.3)	2,571 (3.7)	2,105 (3.3)	.031
**Delayed graft function, Y, No.(%)**		169 (23.4)	8,093 (14.6)	3,524 (12.6)	17,669 (25.4)	13,857 (21.7)	<.001
**Acute rejection, Y, No.(%)**		20 (2.7)	4,919 (8.8)	1,741 (6.2)	4,835 (6.9)	5,553 (8.6)	<.001
**KDPI, mean (SD)**		0.49 (0.26)	0.38 (0.26)	0.42 (0.26)	0.49 (0.26)	0.45 (0.27)	<.001
**Donor age, yrs, mean (SD)**		41.93 (15.52)	37.64 (14.67)	40.90 (14.67)	42.02 (14.86)	39.56 (15.26)	<.001
**Donor BMI, kg/m^2^, mean (SD)**		28.00 (6.60)	26.92 (5.93)	27.10 (5.95)	27.90 (6.40)	27.50 (6.45)	<.001
**Donor creatinine, mg/dl, mean (SD)**		1.15 (1.07)	1.04 (0.83)	1.04 (0.82)	1.15 (1.00)	1.13 (0.94)	<.001
**Donor type, deceased, No.(%)**		515 (70.6)	33,514 (60.3)	16,536 (58.6)	51,921 (74.2)	48,745 (75.8)	<.001
**Donor smoking history, N, No.(%)**		497 (68.2)	40,065 (72.1)	19,886 (70.5)	49,761 (71.1)	45,837 (71.3)	<.001
**Donor hypertension history, Y, No.(%)**		463 (63.5)	32,532 (58.5)	17,073 (60.5)	49,371 (70.5)	39,865 (62.0)	<.001
**Donor DM history, Y, No.(%)**		375 (51.4)	28,709 (51.6)	14,938 (53.0)	42,687 (61.0)	32,946 (51.2)	<.001
**Immunosuppressant** **at discharge, No.(%)**	**IL-2**	218 (29.9)	14,430 (26.0)	7,223 (25.6)	17,776 (25.4)	14,988 (23.3)	<.001
	**T-cell depletion**	417 (57.2)	33,362 (60.0)	17,115 (60.7)	44,006 (62.8)	40,030 (62.2)	<.001
	**CsA**	40 (5.5)	4,099 (7.4)	1,982 (7.0)	3,829 (5.5)	4,314 (6.7)	.045
	**TAC**	423 (58.0)	33,304 (59.9)	16,676 (59.1)	39,381 (56.2)	38,761 (60.3)	.001
	**MPA**	514 (70.5)	39,295 (70.7)	19,858 (70.4)	47,072 (67.2)	45,239 (70.3)	.017
	**mTOR inhibitor**	18 (2.5)	2,136 (3.8)	1,203 (4.3)	2,215 (3.2)	2,716 (4.2)	.015

BMI, body mass index; CsA, cyclosporin A; HLA, human leukocyte antigen; IL-2, IL-2 receptor antibody; M, male; MPA, mycophenolic acid; mTOR, mammalian target of rapamycin inhibitor; PRA, panel-reactive antibody; SD, standard deviation; TAC, tacrolimus; Y, yes; yrs, years. PRA includes candidate peak PRA (before 30/9/2009) or initially calculated PRA (since 30/9/2009).

Regarding donor characteristics, the RCC group had poor deceased donor kidney quality with the highest KDPI score (0.49 [0.26]), the second oldest mean age (41.93 [15.52] years), and highest BMI and creatinine of 28.00 (6.60) kg/m^2^ and 1.15 (1.07) mg/dl, respectively. The percentage of deceased donors was higher in the RCC group than in the GN group (70.6 vs. 60.3%) (**Table 3**).

Regarding immunosuppressive therapy at discharge, only interleukin-2 receptor antibody and T-cell-depleting antibody levels in the RCC group differed significantly from those of the other groups, while the mammalian target of rapamycin inhibitor (mTOR), known for its antioncogenic effects, was used least in patients with RCC-induced ESRD (**Table 3**). At the most recently reported follow-up, the most (n=22 [3%]) recipients were diagnosed with renal cancer after surgery. The mean interval of 4.14 (2.38) years between the transplantation and the renal cancer diagnosis in the RCC group was the shortest of all groups. Additionally, 17.8% (n=130) of other malignancies diagnosed after transplantation among patients with RCC-induced ESRD was the highest. The outcomes of RCC-induced ESRD were the second worst; only 64.2% of those patients survived, slightly more than the diabetes group with the lowest survival rate (62.8%) and highest death rate (30.9%). The major known cause of death of the RCC group was cancer (18.3%), while that of the other groups was cardiocerebrovascular disease (**Table 4**).

**Table 4:** Recipient status at most follow-up with cause of death.

Recipient status, No.(%)		RCC(n = 729)	GN(n = 55,595)	PKD(n = 28,211)	Diabetes(n = 70,020)	Hypertension(n = 64,306)	P-value
**Waiting time, yrs, mean (SD)**		2.24 (1.71)	2.31 (1.86)	2.29 (1.74)	2.39 (1.82)	2.62 (1.99)	<.001
**Renal cancer diagnosed after TX, Y, No.(%)**		22 (3.0)	329 (0.6)	118 (0.4)	290 (0.4)	481 (0.7)	<.001
**Renal cancer diagnosed duration, yrs, mean (SD)**		4.14 (2.38)	5.57 (3.95)	4.73 (4.09)	4.54 (3.28)	4.91 (3.71)	<.001
**Other malignancy diagnosed after TX, No.(%)**		130 (17.8)	3,621 (12.8)	4,617 (8.3)	4,888 (7.0)	4652 (7.2)	<.001
**Follow-up period, yrs, mean (SD)**		5.57 (4.00)	6.22 (4.50)	6.52 (4.67)	4.89 (3.68)	5.65 (4.15)	<.001
**Patient status, No.(%)**	**Alive**	468 (64.2)	39,666 (71.3)	20,976 (74.4)	43,945 (62.8)	42,983 (66.8)	<.001
	**Retransplant**	11 (1.5)	3,641 (6.5)	1,038 (3.7)	1,049 (1.5)	2,446 (3.8)	
	**Lost/Unknown**	32 (4.4)	4,391 (7.9)	2,002 (7.1)	3,422 (4.9)	4,221 (6.6)	
	**Dead**	218 (29.9)	7,897 (14.2)	4,195 (14.9)	21,604 (30.9)	14,656 (22.8)	
**Cause of death, No.(%)**	**Cardiocerebrovascular**	28 (12.8)	979 (12.4)	607 (14.5)	4,074 (18.9)	2,104 (14.4)	<.001
	**infection**	15 (6.9)	831 (10.5)	476 (11.3)	2,631 (12.2)	1,522 (10.4)	
	**Cancer**	40 (18.3)	759 (9.6)	478 (11.4)	1,098 (5.1)	1,071 (7.3)	
	**Graft failure**	2 (0.9)	60 (0.8)	18 (0.4)	104 (0.5)	78 (0.5)	
	**Other/Unknown**	133 (61.0)	5,268 (66.7)	2,616 (62.4)	13,697 (63.4)	9,881 (67.4)	

SD, standard deviation; TX, transplantation; Y, yes; yrs, years.

**Figure 3:**
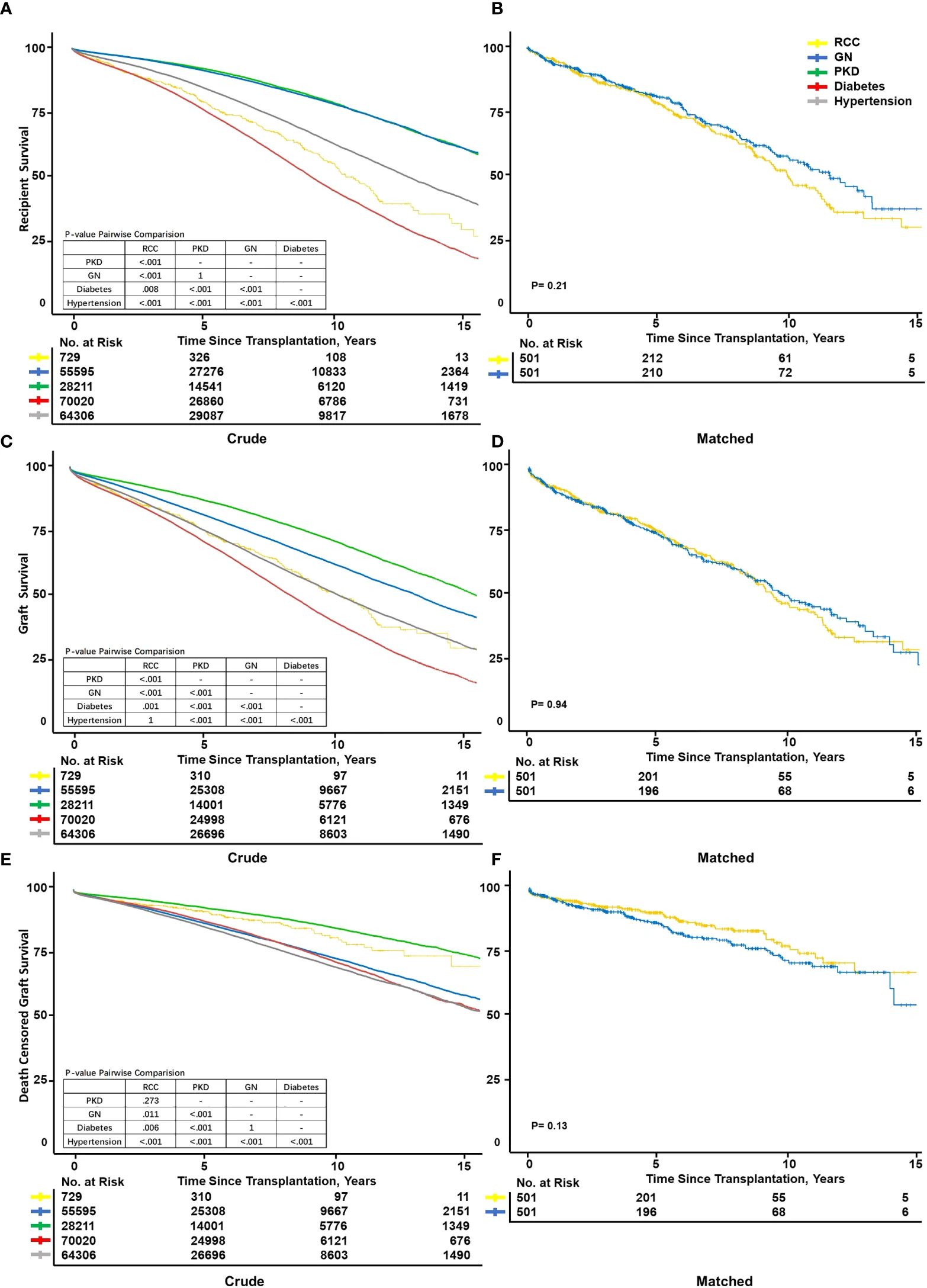
Kaplan–Meier survival curve fit of recipient and graft survival. Kaplan–Meier survival curves showed patient survival between different primary diseases after transplantation before **(A)** and after **(B)** matching. Graft survival crude **(C)** and after matched **(D)**. Death-censored graft survival before **(E)** and after **(F)** propensity score matching. Exact matching variables: Age, ethnicity; not exact: Sex, body mass index, donor age, donor body mass index, donor smoking history, and donor hypertension history.

In the recipient crude survival analysis, the RCC group (79.4 [76–83] %) had the second worst PS, better than only diabetes (75.6 [76.2–77] %), while GN had a significantly optimum PS of 91.5 ([91.2–91.8] %) (**Figure 3A**). The RCC group showed the second worst GS, better than only diabetes (75.7 [72.2–79.4] % and 71.6 [71.2–72] %, respectively) and the second best DCGS, worse than only PKD (91.1 [88.7–93.6] and 92.8 [92.4–93.1] %, respectively) (**Figures 3C,E**). Considering that the RCC group had a much higher mean age than the other groups (8–16 years), the age disparity may had affected the comparison. The PSM of 501 pairs of patients revealed that the PS, GS, and DCGS were not significantly worse in the RCC group than in the GN, which was the best outcome of all groups (p=0.21, p=0.94, and p=0.13, respectively) (**Figures 3B, D, F**). Compared with PKD in PSM, RCC still showed poorer PS (aHR,1.59[1.34-1.87], 77.5 [73.3–82.1] vs. 84.2 [80.4–88.2] %, p=0.029) and GS (73.7 [69.2–78.4] vs. 80.1 [76.1–84.4] %, p=0.039), while comparable DCGS (89.1 [85.9–92.4] vs. 90.3 [87.3–93.5] %, p<0.32) (**Supplementary Figures 1B–D**). Using PKD patients as the control group, we observed a worse outcome in RCC patients both before and after KTX. This is mainly caused by the excelled survival in PKD patients. However, the major cause of ESRD remains to be GN, which has comparable outcome with RCC patients in our study. On the other hand, RCC patients had a 12% hazard reduction (aHR,1.59[1.34-1.87] vs. 1.71[1.51-1.94]) after KTX as compared with remaining on dialysis. This significant benefit deserves to be introduced to RCC patients at consulting.

### Survival benefit: Transplanted versus still waiting in the renal cell carcinoma group

To explore whether RCC-induced ESRD patients could benefit from KTX, we compared those who received transplantation with those who were still waiting for transplantation. Compared with the still-waiting patients, those who received transplantation were younger (59.1 vs. 61.3 years), had a lower proportion of obesity (36.2% vs. 41.6%) and diabetes (13.3% vs. 24.1%), and had a longer dialysis history (77.6% vs. 69.1%). The major cause of death among the transplanted patients was cancer, while that among the still-waiting patients was cardiocerebrovascular disease (48.2% and 61.8%, respectively) (**Table 5**).

**Table 5:** Renal cell carcinoma (RCC) patients’ characteristics and cause of death at most recent follow-up.

Characteristics		Transplanted (n = 729)	Still Waiting (n = 818)	p- value
**Age, yrs, mean (SD)**		59.1 (11.3)	61.3 (9.9)	<.001
**Sex, M, No.(%)**		549 (75.3)	619 (75.7)	.915
**Ethnicity, No.(%)**	**White**	509 (69.8)	528 (64.5)	.079
	**Black**	158 (21.7)	192 (23.5)	
	**Hispanic**	46 (6.3)	70 (8.6)	
**BMI ≥ 30 kg/m^2^, Y, No.(%)**		264 (36.2)	340 (41.6)	.036
**Diabetes, Y, No.(%)**		97 (13.3)	197 (24.1)	<.001
**Education level, college degree, No.(%)**		273 (37.4)	300 (36.7)	.793
**Private health insurance, No.(%)**		393 (53.9)	432 (52.8)	.732
**Dialysis, Y, No.(%)**		566 (77.6)	565 (69.1)	<.001
**Death, No.(%)**		83(11.4)	55(6.7)	<.001
**Cause of death,** **No.(%)**	**Cardiocerebrovascular**	28 (33.7)	34 (61.8)	.003
	**Infection**	15 (18.1)	9 (16.4)	
	**Cancer**	40 (48.2)	12 (21.8)	

BMI, body mass index; M, male; RCC, renal cell carcinoma; SD, standard deviation; Y, yes; yrs, years.

**Figure 4:**
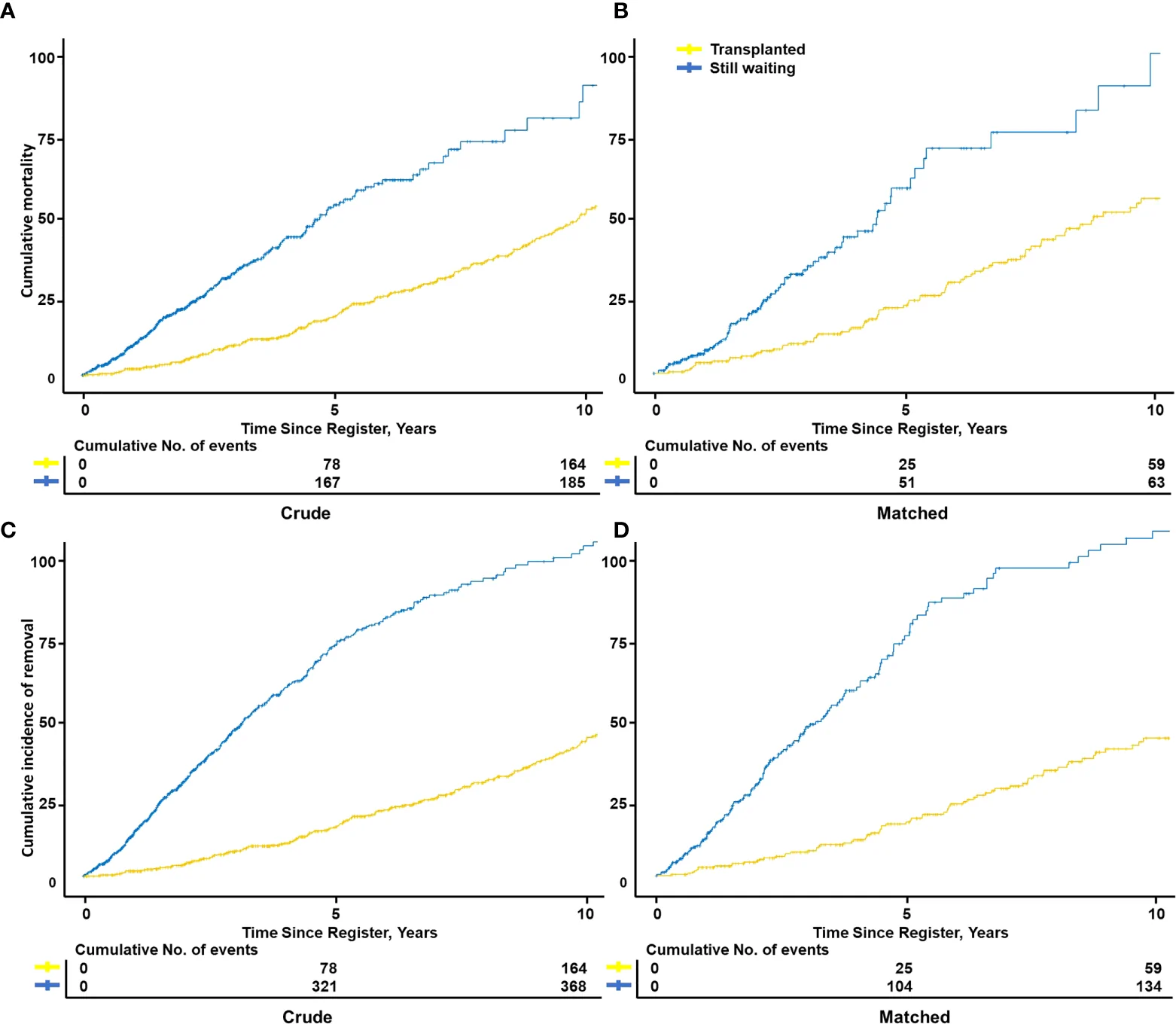
Cumulative mortality or removal incidence of transplanted vs. still waiting among RCC-induced ESRD patients. Kaplan–Meier curves showed patient cumulative mortality between transplanted and still-waiting patients from register to death, both before **(A)** and after (n=323) matching **(B)**. Considered some still-waiting patients were removed due to death or deterioration, the patient cumulative removal incidence from the registration to removal of two groups was compared both before **(C)** and after **(D)** matching. Matching variables: Age, body mass index, sex, ethnic, diabetes history, primary insurance, education level.

The transplanted group showed a significantly better outcome than the still-waiting group, both before (12.8 [10.1–15.5] vs. 37.8 [32.2–43.0] %) and after PSM (13.6 [9.3–17.8] vs. 38.4 [28.3–47.1] %) (**Figures 4A, B**). Unsurprisingly, after adding deterioration to the removal incidence, the advantage between two groups was further expanded before (12.8 [10.1–15.5] vs. 61.7 [56.6–66.2] %) and after PSM (13.6 [9.3–17.8] vs. 61 [52–68.4] %, respectively) (**Figures 4C, D**).

Furthermore, we used Cox regression analysis to calculate the HR of the transplanted versus still-waiting patients in the RCC, GN, and PKD groups, respectively. The KTX patients had an 84% increased removal risk, similar with that of the GN and PKD patients (aHR, 0.16 [0.13–0.20] vs. 0.16 [0.14–0.18]) (**Table 6**).

**Table 6:** Cox regression of RCC versus glomerulonephritis versus polycystic kidney disease for estimating survival benefit from transplantation.

Group	Incident	Unadjusted Hazard Ratio^a^ (95%CI)	Adjusted^b^Hazard Ratio (95%CI)
**RCC**	Death	0.34 (0.28-0.43)	0.32 (0.26-0.41)
	Removal	0.17 (0.14-0.21)	0.16 (0.13-0.20)
**GN**	Death	0.26 (0.23-0.30)	0.27 (0.23-0.31)
	Removal	0.16 (0.14-0.18)	0.16 (0.14-0.18)
**PKD**	Death	0.21 (0.20-0.22)	0.23 (0.22-0.25)
	Removal	0.12 (0.12-0.13)	0.13 (0.13-0.14)

a Still-waiting group as a reference.

b Adjusted for whether transplanted, age, sex, ethnicity, BMI, primary insurance, diabetes history, and dialysis before transplantation.

CI, confidence interval; GN, glomerulonephritis; RCC, renal cell carcinoma; PKD, polycystic kidney disease.

### Donor-type differences: Living donor versus donation after circulatory death versus donation after brain death

To highlight the differences with regard to the donor type, we separated the RCC group into living donor (LD), donation after circulatory death (DCD), and donation after brain death (DBD) to process a subgroup comparison. Among the RCC recipient cohorts, LD patients were more Whites, more likely to have private health insurance and high education level, less likely to have dialysis history and donor hypertension history, less likely to use IL-2 receptor antibody and more likely to use T-cell depletion. DCD patients were more Blacks, more likely to have dialysis history, donor hypertension and diabetes history, more likely to use IL-2 receptor antibody and less likely to use T-cell depletion, tacrolimus and mycophenolic acid (MPA). DBD patients were more tacrolimus, MPA, and mTOR using. In respect of the patient status, DBD recipients suffered a horribly higher death rate than LD or DCD (26.2% vs. 9.3% vs. 9.7%). The major known cause of death of the LD and DCD was cancer (60% and 50%, respectively), while that of DBD was cardiocerebrovascular disease (47.2%) (**Supplementary Table S1**). Regarding outcomes, LD recipients were better than deceased donor recipients, while DCD and DBD were not significantly different. To note, three types of RCC patients were all worse than PKD patients (**Supplementary Figures 2A–C**). Furthermore, compared with patients who are still waiting, the patients who received LD could benefit more from KTX than DCD, while the patients who received DBD could benefit less (aHR, 0.29[0.20-0.42] vs. 0.31[0.19-0.50] vs. 0.36[0.28-0.46]) (**Supplementary Table S2**).

## Discussion

The RCC-induced ESRD population may be too small to attract researchers’ interest, so we designed a 20-year retrospective cohort study of 1,547 patients. The study demonstrated that RCC-induced ESRD patients suffered from relative frailty and an unfulfilling economic and social background. Despite these poorer baseline characteristics, RCC-induced ESRD patients still presented favorable outcomes similar to GN patients. Furthermore, RCC-induced ESRD patients considerably benefited from the favorable outcomes of transplantation.

RCC-induced ESRD patients were older, more likely obese, more likely diabetic, more likely to have poor kidney function, and less likely to have private health insurance, which indicates a relatively unfulfilling socioeconomic status. RCC is a male-predominant (2:1 ratio) disease with a typical presentation in 60-year-olds (17, 18). Our study also found that the age and sex of RCC candidates and recipients were similar with those of the general RCC population. The transplantation outcome depends on patient demographic characteristics, comorbidity, and competing risks of mortality. Frailty has been consistently associated with worse outcomes after surgery for RCC (19).

Unsurprisingly, the highest post-transplant renal cancer incidence and shortest interval between renal cancer diagnosis and transplantation were found in the RCC group. In fact, the 3% recurrence rate of renal cancer at the most recently reported follow-up in this study was higher than that of the general population isolated a local rare recurrence incidence of 1%–2% at 5-year follow-up (20–23). According to previous studies, the patient’s age, sex, race, and BMI did not significantly influence cancer recurrence (24). However, pre-TM was proven associated with the development of post-transplant malignancy; in fact, the 19% of pre-TM recipients who died of malignancy was consistent with the 18.3% RCC-induced ESRD patients in our study (25). Post-transplant malignancy as major cause of death still overwhelmingly impaired patient survival. However, unlike patients with diabetes and hypertension, who suffered both poor patient survival and poor GS, RCC-induced ESRD patients had relatively good GS. RCC recurrence might impair patient lifespan but would not continue to the damage the allograft, as approximately 80%–90% of renal cancers occur in native kidneys (26–28). Moreover, a surgical approach that can influence the clinical prognosis of these patients should be considered. Recent research underlines that robotic surgery in patients with RCC undergoing partial nephrectomy had better long-term oncological outcomes including higher overall survival, lower local recurrence, and a metastatic disease incidence rate, even a renal functional outcome with less chronic kidney disease upstaging, compared with laparoscopic and open surgery (29).

In previous studies, RCC-induced ESRD patients were evaluated carefully using strict strategies, and a delayed waiting time was required to prevent cancer recurrence (11, 13–15) out of most clinicians’ worries about immunosuppressant use (30, 31). Although the transplant rate of RCC-induced ESRD patients was not worse than that of non-cancer-caused ESRD patients in the UNOS, poorer-quality donors, which might lead to PNF and/or DGF and a longer preoperative dialysis duration, might impair patient survival (32), indirectly illustrating transplantation discrimination. Combined with poor health condition and limited personal medical resources, these disadvantages of RCC-induced ESRD patients result in worse patient survival than that of GN recipients. Although the waiting time of the RCC group was not longer than that of the other groups, medical staff may hesitate to enroll RCC patients on the waiting list. Therefore, the waiting time of RCC-induced ESRD patients before being listed could be indicated better by the dialysis duration. The RCC group had a 1.5-year longer dialysis duration before KTX compared with the GN group. However, after the elimination of these disadvantages, RCC-induced ESRD recipients showed favorable outcomes. Coupled with a previous study, a delay after curative treatment did not appear to protect against cancer recurrence (33). Even if contraindication and a delayed waiting time of transplantation were not encouraged, preoperative frailty and oncology assessment to identify patients who are expected to benefit most from transplantation, aiming to optimize decision-making and postoperative outcomes in RCC-induced ESRD patients, are needed (19).

Furthermore, RCC-induced ESRD patients had a distinctly improved survival rate after transplantation compared to those still waiting, and the extent of the benefit from transplantation was paralleled to that of non-cancer-caused ESRD patients. Both the OPTN and the Danish analysis suggested that transplant recipients had a 39%–70% lower risk of death compared to that of 65-year-old or older patients who remained on dialysis therapy (34, 35). Furthermore, Chaudhry et al. reported that transplantation was associated with a significant reduction in long-term mortality risk compared with waitlisted patients on dialysis with kidney failure (36). The evidence from our study also found that KTX could overwhelmingly decrease (by 84%) the risk of death or deterioration among RCC-induced ESRD patients.

However, the current study has several potential limitations. First, immunosuppressant use is among the most important factors due to its influence on the cancer. Nevertheless, the immunosuppressant regimen used during follow-up combined with the many confounders and potential for bias in the candidate selection process for renal transplantation are insufficiently detailed in the UNOS registry. Second, a lack of granularity of the data, particularly as it relates to RCC details such as grade, stage, and time from surgery, the study fails to distinguish whether post-transplantation cancer is occurrence or recurrence and RCC or another pathologic type. Although post-transplantation renal cancer was selected from among *de novo* renal cancers, it remains difficult to distinguish renal carcinoma in a native kidney versus that in an allograft. Third, considering that UNOS data were only available across the United States, a total of 43,997 recipients who underwent transplantation outside of the United States were missed, which may result in the underestimation of the transplant rate. Moreover, pretransplant RCC, including patients diagnosed with cancer after being listed and ESRD caused by other primary diseases despite an RCC history, was not investigated in this study.

In conclusion, RCC-induced ESRD patients who underwent KTX presented a good prognosis and could considerably benefit from transplantation in terms of patients and GS. Hence, these patients should not be limited to transplantation by strict strategies or a delayed waiting time out of their malignancy history. Further studies are necessary to clarify the risk factors affecting the transplant outcomes of patients with RCC-induced ESRD versus those of all pretransplant RCC patients.

## Data availability statement

Publicly available datasets were analyzed in this study (https://optn.transplant.hrsa.gov/data/request-data/). The authors declare that the data supporting the findings of this study are available and will be provided upon request.

## Author contributions

XH, WL, QY, and JD designed the study. XH and WL conducted statistical analysis and wrote the manuscript. All authors contributed to the critical revision of the manuscript for intellectual content and revised the manuscript. All authors read and approved the final manuscript.

## Acknowledgments

We gratefully acknowledge research support of the OPTN/UNOS data and guiding the data analysis provided by Dr. Nahel Elias of the Center for Transplantation Sciences and Division of Transplant Surgery, Department of Surgery, Massachusetts General Hospital, Boston, MA, USA.

## Conflict of interest

The authors declare that the research was conducted in the absence of any commercial or financial relationships that could be construed as a potential conflict of interest.

## Correction note

A correction has been made to this article. Details can be found at: 10.3389/fonc.2026.2000045.

## Publisher’s note

All claims expressed in this article are solely those of the authors and do not necessarily represent those of their affiliated organizations, or those of the publisher, the editors and the reviewers. Any product that may be evaluated in this article, or claim that may be made by its manufacturer, is not guaranteed or endorsed by the publisher.

## Supplementary material

Supplementary Figure 1 Kaplan Meier survival curve fit of candidate cumulative removal incidence, recipient and graft survival of RCC versus PKD. Comparison of survival status between RCC group and PKD group before and after transplantation. Before transplantation, Candidate survival status was shown by cumulative removal incidence. RCC group and PKD group were matched by exact matching variables: Age, sex, ethnicity, diabetes history and education level; and not exact variables: Body mass index, primary insurance and income and then got 868 pairs of patients. **(A)** After transplantation, recipient survival status was shown by PS, GS and DCGS. RCC group and PKD group were matched by exact matching variables: Age, ethnicity; and not exact: Sex, body mass index, donor age, donor body mass index, donor smoking history, and donor hypertension history. **(B–D)**.

Supplementary Figure 2 Kaplan Meier survival curve fit of RCC recipient outcome in three donor types. Comparison of outcome between three types of RCC group after transplantation. **(A)** patient survival; **(B)** graft survival; **(C)** death censored graft survival. PKD group was set as a reference in the figure. RCC, renal cell carcinoma; LD, living donor; DCD, donation after circulatory death; DBD, donation after brain death; PKD, polycystic kidney disease.

Supplementary Table 1 Baseline and status of three types of RCC recipients and donors. LD, living donor; DCD, donation after circulatory death; DBD, donation after brain death; BMI, body mass index; CsA, cyclosporin A; IL-2, IL-2 receptor antibody; M, male; MPA, mycophenolic acid; mTOR, mammalian target of rapamycin inhibitor; SD, standard deviation; TAC, tacrolimus; Y, yes; yrs, years.

Supplementary Table 2 Cox regression of RCC living donor versus donation after circulatory death versus donation after brain death for estimating survival benefit from transplantation. ^a^Still-waiting group as a reference. ^b^Adjusted for whether transplanted, age, sex, ethnicity, BMI, primary insurance, diabetes history, and dialysis before transplantation. CI, confidence interval; LD, living donor; DCD, donation after circulatory death; DBD, donation after brain death.

## Abbreviations

aHR, adjusted hazard ratio; BMI, body mass index; DCGS, death- censored graft survival; ESRD, end-stage renal disease; GN, glomerulonephritis; OPTN, Organ Procurement and Transplantation Network; PKD, polycystic kidney disease; RCC, renal cell carcinoma; UNOS, United Network for Organ Sharing.
