# Correction: Transplant or dialysis: what’s the better choice for RCC-induced ESRD patients? A 20-year analysis of OPTN/UNOS data

**DOI:** 10.3389/fonc.2026.2000045

**Published:** 2026-09-28

**Authors:** Xiaowei Hao, Wenhui Lai, Xinze Xia, Junnan Xu, Yangyang Wu, Chao Lv, Kaikai Lv, Shuai Huang, Zhenjun Luo, Qingyang Meng, Qing Yuan, Jun Dong

**Affiliations:** 1 Department of Urology, The Third Medical Center, Chinese PLA General Hospital, Beijing, China; 2 Department of Urology, No.971 Hospital of PLA Navy, Tsingtao, Shandong, China; 3 Department of Postgraduate, Hebei North University, Zhangjiakou, China; 4 Department of Urology, Shanxi Medical University, Taiyuan, China; 5 Affiliated Hospital of Weifang Medical University, School of Clinical Medicine, Weifang Medical University, Weifang, China

**Keywords:** renal cell carcinoma, kidney transplantation, end-stage renal disease, UNOS/OPTN, propensity score match

Affiliation 2, “Department of Urology, No.971 Hospital of PLA Navy, Tsingtao, Shandong, China,” was omitted from the paper. This affiliation has now been added for author “Xiaowei Hao”.

The original version of this article has been updated.

