# Supplementary figures and images for: Transplant or dialysis: What’s the better choice for RCC-induced ESRD patients? A 20-year analysis of OPTN/UNOS data
Source: Front Oncol. 2022 Sep 29;12:955771. doi: 10.3389/fonc.2022.955771 (PMC9558276; doi:10.3389/fonc.2022.955771)

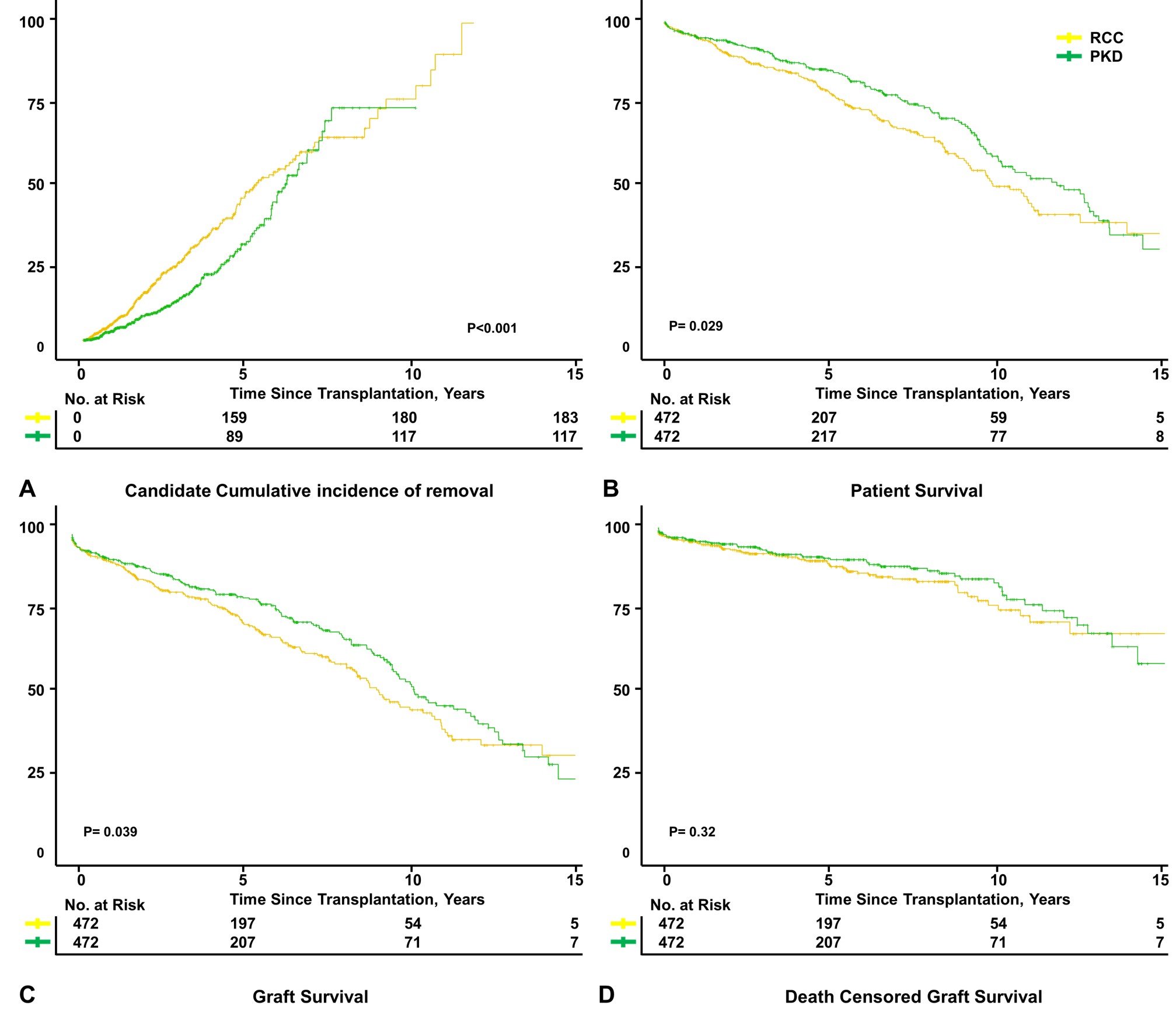

Supplement: Supplementary Figure 1 — Kaplan Meier survival curve fit of candidate cumulative removal incidence, recipient and graft survival of RCC versus PKD. Comparison of survival status between RCC group and PKD group before and after transplantation. Before transplantation, Candidate survival status was shown by cumulative removal incidence. RCC group and PKD group were matched by exact matching variables: Age, sex, ethnicity, diabetes history and education level; and not exact variables: Body mass index, primary insurance and income and then got 868 pairs of patients. (A) After transplantation, recipient survival status was shown by PS, GS and DCGS. RCC group and PKD group were matched by exact matching variables: Age, ethnicity; and not exact: Sex, body mass index, donor age, donor body mass index, donor smoking history, and donor hypertension history. (B–D). [file Image_1.jpeg]

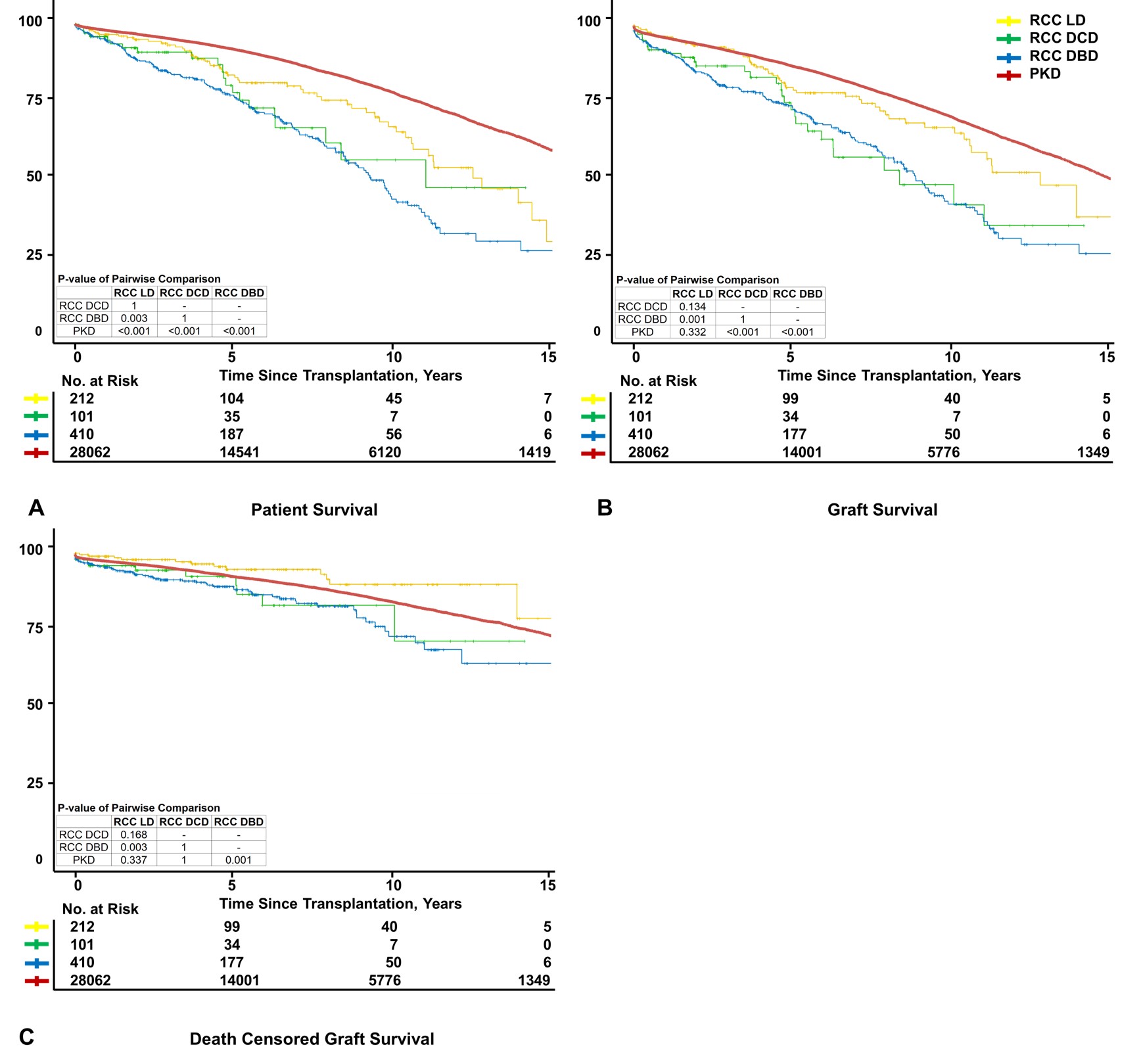

Supplement: Supplementary Figure 2 — Kaplan Meier survival curve fit of RCC recipient outcome in three donor types. Comparison of outcome between three types of RCC group after transplantation. (A) patient survival; (B) graft survival; (C) death censored graft survival. PKD group was set as a reference in the figure. RCC, renal cell carcinoma; LD, living donor; DCD, donation after circulatory death; DBD, donation after brain death; PKD, polycystic kidney disease. [file Image_2.jpeg]
